# Obstacles to the uptake of breast, cervical, and colorectal cancer screenings: what remains to be achieved by French national programmes?

**DOI:** 10.1186/1472-6963-14-465

**Published:** 2014-10-04

**Authors:** Jonathan Sicsic, Carine Franc

**Affiliations:** Cermes3, UMR8211, Inserm U988, Site CNRS, 7, rue Guy Moquet, 94801 Villejuif Cedex, France

**Keywords:** Breast cancer screening, Cervical cancer screening, Colorectal cancer screening, Inequalities

## Abstract

**Background:**

In France, equality in access to screening has been one of the main thrusts of public policies implemented between 2009 and 2013 (the national cancer plan). Our aim in this study was to analyse the obstacles to and levers for breast, cervical, and colorectal cancer screening uptake and their trends over time.

**Methods:**

Based on representative data from the French Health Care and Health Insurance Survey (three independent, cross-sectional surveys: 2006, 2008, and 2010), multivariate logistic regressions were used to model the association between the nonuse of screening for the three cancers and various independent variables. Then, interactions with survey year dummies allowed the changes in the determinants of these cancer screenings over time to be estimated.

**Results:**

Whereas the incentives for screening were strengthened during the period considered, cervical and breast cancer screenings decreased, and colorectal cancer screenings increased sharply (from 18.2% (95% CI = [17.0-19.4]) in 2006 to 38.9% (95% CI = [37.4-40.5] in 2010. Under-users of the three cancer screenings were primarily unskilled workers (OR_cervix_ = 1.64 [1.38-1.95]), individuals without complementary health insurance (OR_breast_ = 2.05 [1.68-2.51]), or individuals with free complementary health insurance who more rarely use outpatient care. Moreover, individuals reporting either risky behaviours, namely heavy smokers (OR_colorectal_ = 1.70) and high-risk drinkers (OR_cervix_ = 1.42) or very safe behaviours, namely neither smoking nor drinking, underused screenings. Despite the implementation of national programmes for breast and colorectal cancer screenings, the disparities and inequalities in screening uptake did not decrease over the study period.

**Conclusions:**

These results demonstrate the need for additional primary prevention efforts targeting the identified under-users by focusing on, for instance, individuals with a very healthy lifestyle. Health authorities could also intensify their efforts to promote increased access to screening for the most disadvantaged individuals.

## Background

Cancer is one of the leading causes of disease and mortality [1]. Breast and colorectal cancer are among the most commonly diagnosed cancers worldwide [2]. Studies published in the mid-1990s have shown that prevention practices and cancer screening both play a key role in reducing cancer prevalence, morbidity, and mortality [3–5]. A recent report from the French National Cancer Institute [6] reaffirms this finding and highlights the central role that primary care physicians play in providing cancer screening. However, recent analyses of the inequalities in screening uptake in France as well as other northern countries are in general agreement, highlighting the persistent role of socioeconomics (e.g., income, educational level, private health insurance access) and family structure [7–11]. For instance, concerning breast and cervical cancer, poor, unmarried women with lower levels of education and less access to health care are less likely to be tested [11, 12]. Beck and Gautier [13] emphasise the positive impact of being older, living in a couple, having children, and more frequently consulting a general practitioner (GP) on colorectal cancer screening uptake. Kobayashi et al. highlight health literacy and Lo et al. highlight dislike of the test as barriers to colorectal cancer screening in England [14] (and Great Britain [15], respectively).

Policy mechanisms have been implemented based on these findings, and cancer screening remains one of the main thrusts of the French *2009*–*2013 Cancer Plan*, with a stated priority of combating inequalities in access to and use of screening. There is no national programme for cervical screening (only experiments are ongoing), but national screening programmes targeting breast cancer and colorectal cancer have been established in France since 2004 and 2009, respectively. The national screening programme for breast cancer targets women between 50 and 74 years of age and consists of sending a voucher through the mail that allows each eligible woman to benefit from a free screening at a radiological centre participating in the programme. The national screening programme for colorectal cancer targets men and women between 50 and 74 years of age and consists of sending a faecal occult blood test (FOBT) (accompanied by directions on how to perform the test) through the mail, for a free laboratory analysis. In addition to these programs, incentives directed towards GPs have been implemented in France, targeting breast cancer screening since 2009 and cervical cancer screening since 2012. These incentives consist of financial bonuses to referral GPs who have screened 80% of their eligible patients. Yet, the impact of these schemes has not been evaluated thus far.

Numerous studies have examined the determinants of cancer screening uptake, but few studies have analysed how these determinants have changed over time, particularly in response to the recent implementation of mass screenings and/or national programmes. The results of preliminary studies indicate that in France, the impact of the social gradient seems to have decreased in 2010 (*vs.* 2005); however, the inequalities in access for the poorest individuals have persisted [16]. In a different context, De Maio et al*.* [17] show that the impact of income and educational level on cervical cancer screening use remained stable between 2005 and 2010 in Argentina, but in the case of breast cancer, the impact of education has decreased.

In the context of the strengthening of prevention policies through the implementation of national screening programs, financial incentives, and a third National Cancer Plan, one would expect the screening rates for breast and colorectal cancers to have increased and the inequalities related to socioeconomics and/or health care access to have decreased between 2006 and 2010. The objective of this paper is to model the determinants of the failure to participate in cervical, breast, and colorectal cancer screenings in France and to study the trends in these determinants over time, i.e., between 2006 and 2010. The goal is twofold: first, to contribute to the debate on the efficacy of policies implemented to reduce screening disparities and/or inequalities and, second, to identify potential remaining obstacles to screening to better target and tailor policies promoting screening use. Throughout the paper, the term "inequalities" refers to observed disparities in cancer screening uptake that are assumed to arise from access to care or systemic barriers. The term "differences" refers to the associations between other variables/determinants (e.g., socioeconomic status, risky behaviours) and cancer screening uptake, for which determining whether they are related to the choices of patients or the equity of the health care system is not possible.

## Methods

### The ESPS survey

The Health Care and Health Insurance Survey (*Enquête Santé et Protection Sociale,* ESPS) was conducted by the Research and Information Institute for Health Economics (IRDES) annually between 1988 and 1997 and has been conducted every 2 years since 1998. The ESPS is administered to a representative, population-based sample of French households that are randomly drawn from public health insurance files. It combines interviews (telephone or face to face) and self-administered questionnaires [18]. The declarative data provide information on both the household (income, household type, region of residence) and its members (e.g., socioeconomic characteristics, health care, and prevention behaviours). All of the beneficiaries of health insurance belonging to the ESPS sample, as well as members of their households, were surveyed every four years. The sampling database was renewed in 2010; thus, the 2006, 2008 and 2010 ESPS samples comprised different subpopulations*.* The response rates were 63% in 2006, 65% in 2008, and 66% in 2010.

### Ethics

This study was planned as a research project. All precautions were taken by the IRDES to ensure anonymity of the data. The construction of the ESPS samples as well as data analysis was approved by the CNIL (Commission Nationale de l’Informatique et des Libertés, French law no. 78–17, authorisation number: 1147702)^a^. According to the French law, written informed consent was not required for this type of study. The three ESPS databases are available upon request and research collaboration with the IRDES.

### Dependent variables

We were interested in 3 outcomes: cervical, breast, and colorectal cancer screenings following the French Health Authority’s guidelines. The first outcome of interest was whether a woman between 25 and 65 years old received a Pap smear for cervical cancer within the past 3 years. The second outcome of interest was whether a woman aged between 50 and 74 years received a mammogram for breast cancer in the past 2 years. The third outcome of interest was whether an individual aged between 50 and 74 years performed a FOBT for colorectal cancer in the past two years.

### Independent variables

The determinants of the cancer screening uptake were grouped into 3 categories: (1) socioeconomic characteristics, (2) health and health care consumption, (3) risky behaviours and prevention use. For category 1, we considered age, region of residence, social class (following the French National Institute of Statistics and Economic Studies codification), marital status, and complementary health insurance status (private/free/no)^b^. In category 2,a dichotomous variable indicating whether the individual reported having a chronic disease (yes/no) was used, and individuals’ opinion of their health was measured with a 4-category, self-rated health variable constructed from a 10-point Likert scale (0 = lowest health state, 10 = best health state). Health care consumption was measured based on the number of consultations with a GP over the past 12 months and the number of consultations with a specialist over the past 12 months. In category 3, risky behaviours were measured via the levels of tobacco and alcohol consumption. Four classes were defined to describe risky behaviours related to tobacco use: nonsmokers, ex-smokers, light smokers (i.e., smoking 10 or fewer cigarettes per day), and heavy smokers (i.e., smoking more than 10 cigarettes per day). Similarly, four alcoholism profiles were constructed following a well-established typology based on the Alcohol Use Disorders Identification Test (AUDIT-C) questionnaire [19, 20]: nondrinkers, safe consumers, occasionally risky consumers, and high-risk consumers. To address prevention use, we included screening activity for other cancers for which individuals were eligible. Thus, in the model for individual breast cancer screening uptake, we included whether the woman had received a FOBT in the past 2 years as a predictor, and in the model for individual colorectal cancer screening (females only), we included whether the woman had received a mammogram in the past 2 years as a predictor^c^.

### Descriptive statistics

The descriptive statistics were used, first, to assess the levels and trends in cervical, breast and colorectal cancer screening uptake and, second, to examine whether the distributions of the independent variables were similar across the surveys.

### Analytical strategy

The three ESPS samples (2006, 2008, and 2010) were pooled, and a multivariate logistic model was estimated to determine the association between the failure to obtain a screening for each of the three cancers with the independent variables, thereby enabling us to highlight persistent differences in screening behaviours. Because all three screenings are applicable to women but only one (colorectal) is applicable to men, separate analyses were conducted by sexe^d^. Two distinct models were estimated for breast cancer screening (colorectal cancer screening): model 1 did not include the FOBT (mammogram) variable as a predictor; whereas model 2 did. Because the inclusion of these variables was suspected to introduce bias into the models, we choose to report the results of model 1. Specific categories of nonresponse were created for each independent variable and included in the models, but we do not report their results, as we were not interested in their effects.

Then, to analyse the changes in the determinants of the failure to obtain screenings over time, we analysed the interactions between the determinants of interest (e.g., age, social class, complementary health insurance, risky behaviours) and survey year dummies (i.e., 2008 *vs.* 2006 and 2010 *vs.* 2006 for the breast cancer and cervical screening models and 2010 *vs.* 2008 for the colorectal cancer screening model), and in the final model, we included the interactions that were statistically significant at the 5% level (selected through backward deletion).

## Results

### Descriptive statistics

**Figure 1:**
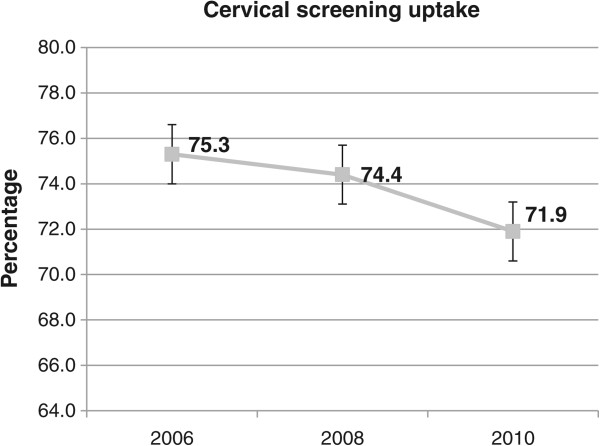
**Trends in cervical screening uptake between 2006 and 2010.**

**Figure 2:**
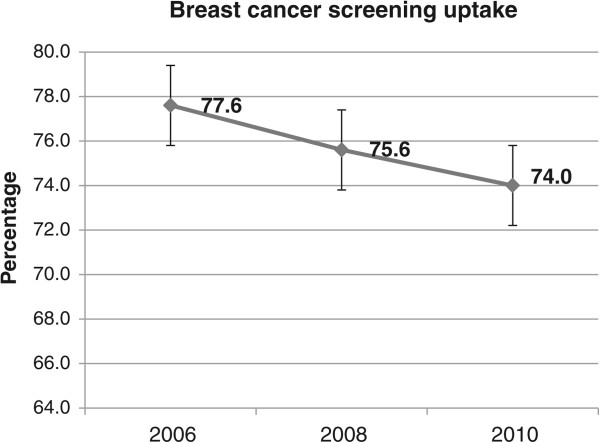
**Trends in breast cancer screening uptake between 2006 and 2010.**

**Figure 3:**
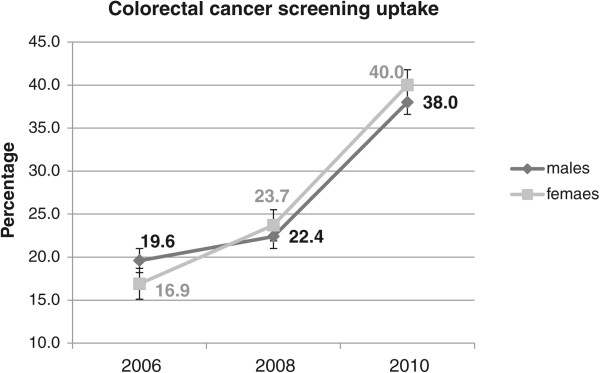
**Trends in colorectal cancer screening between 2006 and 2010.**

The results regarding the trends in cervical, breast, and colorectal cancer screenings between 2006 and 2010 are displayed in Figure 1, Figure 2 and Figure 3.

Whereas 75.3% (95% CI = [74.1%-76.5%]) of women reported having received a test for cervical cancer in 2006, this rate decreased significantly to 71.9% (95% CI = [70.5%-73.3%]) in 2010. The screening rate for breast cancer decreased (but not significantly at the 5% level) between 2006 and 2010, from 77.6% (95% CI = [75.8%-79.3%]) in 2006 to 74.0% (95% CI = [72.1%-75.9%]) in 2010. Overall, colorectal cancer screening uptake increased significantly between 2006 and 2010, from 18.2% (95% CI = [17.0%-19.4%]) in 2006 to 38.9% (95% CI = [37.4%-40.5%] in 2010. As shown in Figure 3, the increase was sharper for women, whose screening uptake increased from 16.9% (*vs.* 19.6% for males) in 2006 to 40.0% (*vs.* 38.0% for males).

Table 1 presents the distribution of the independent variables according to the survey year. For most determinants of cancer screening uptake, the proportion of respondents in the various categories did not change across the surveys. The main exceptions are having free health insurance (9.7% in 2010 *vs*. 6.5% in 2006), having a chronic disease (58.5% in 2010 *vs*. 68.8% in 2006), having seen a specialist a least once (47.1% in 2010 *vs*. 42.0% in 2008) and never drinking alcohol (19.3% in 2008 *vs*. 24.2% in 2010).

**Table 1:** **Descriptive statistics: the distribution of the independent variables in the three ESPS surveys**

	2006		2008		2010	
Determinants of cancer screening	N	%	N	%	N	%
	Socioeconomic characteristics					
**Age**						
[25–49]	5876	56.1	5205	53.2	4825	54.0
[50–65]	3404	32.5	3372	34.5	3092	34.6
[66–74]	1203	11.5	1209	12.4	1021	11.4
**Housing region**						
Ile de France (without Paris)	1574	15.0	1350	13.8	1256	14.1
Paris and suburbs	1870	17.8	1895	19.4	1614	18.1
North	741	7.1	709	7.3	657	7.4
Est	958	9.1	851	8.7	795	8.9
West	1500	14.3	1443	14.8	1511	16.9
South West	1277	12.2	1134	11.6	1030	11.6
Centre	1468	14.0	1279	13.1	1143	12.8
Mediterrannée	1095	10.5	1125	11.5	914	10.3
**Social class**						
Farmer	353	3.4	302	3.1	259	2.9
Craftsman	581	5.5	595	6.1	521	5.8
Manager	1359	13.0	1291	13.2	1201	13.4
Associate prof.	2159	20.6	2097	21.4	1666	18.6
Office worker	2904	27.7	2761	28.2	2561	28.7
Skilled worker	1766	16.9	1572	16.1	1491	16.7
Nonskilled worker	1046	10.0	939	9.6	880	9.9
Inactive	301	2.9	215	2.2	316	3.5
**Marital status**						
Living in a couple	8425	80.4	7805	79.8	7142	79.9
Single	1115	10.6	1049	10.7	978	10.9
Nonresponse	943	9.0	932	9.5	818	9.2
**Complementary health insurance**						
Private	9150	87.3	8628	88.2	7721	86.4
Free	682	6.5	674	6.9	868	9.7
No	618	5.9	454	4.6	300	3.4
	**Health and health care consumption**					
**Chronic disease**						
Yes	2610	24.9	2561	26.2	3196	35.8
No	7217	68.8	6574	67.2	5227	58.5
Don't know	656	6.3	651	6.7	515	5.8
**Self-rated health**						
[0–4] : poor	365	3.5	403	4.1	386	4.3
[5, 6] : fair	1604	15.3	1709	17.5	1362	15.2
[7, 8] : good	4870	46.5	4699	48.0	4090	45.8
[9, 10] : very good	3225	30.8	2555	26.1	2688	30.1
Nonresponse	419	4.0	420	4.3	412	4.6
**Nb of consultations with a GP**						
Two or less	4884	46.6	4539	46.4	4133	46.2
Three or more	4983	47.5	4693	48.0	4208	47.1
Nonresponse	616	5.9	554	5.7	597	6.7
**Nb of consultations with a specialist**						
None	4154	39.6	3690	37.7	3302	36.9
At least one	4578	43.7	4112	42.0	4208	47.1
Nonresponse	1751	16.7	1984	20.3	1428	16.0
	**Risky behaviours**					
**Tobacco consumption**						
Non-smoker	3608	34.4	3434	35.1	2992	33.5
Ex-smoker	2825	27.0	2562	26.2	2101	23.5
Light smoker	1357	12.9	1412	14.4	1495	16.7
Heavy smoker	1428	13.6	1241	12.7	1161	13.0
Nonresponse	1265	12.1	1137	11.6	1189	13.3
**Alcohol consumption**						
Non drinker	2234	21.3	1893	19.3	2164	24.2
Safe consumer	4590	43.8	4338	44.3	3676	41.1
Occasionnaly risky consumer	2575	24.6	2288	23.4	1997	22.3
High risk consumer	531	5.1	748	7.6	629	7.0
Nonresponse	553	5.3	519	5.3	472	5.3

### Multivariate analyses

#### Cervical screening

The results of the main-effects and interactions models for cervical screening are displayed in Table 2 (only one interaction was significant at the 5% level).

**Table 2:** **Multivariate logistic regression models of the absence of cervical screening (N = 13,177 women)**

Independant variables	No cervical screening ***main effects model***	No cervical screening ***interactions model***
	OR (95 CI)	OR (95 CI)
	Socioeconomic characteristics	
**Age**	*(ref = 25–34)*	
[35–44]	1.05 (0.92-1.20)	1.01 (0.81-1.25)
[45–54]	1.56*** (1.37-1.78)	1.19 (0.97-4.47)
[55–65]	2.39*** (2.08-2.74)	2.05*** (1.65-2.56)
Age *survey year	*(ref: age = '25-34'; survey year = '2006')*	
[45–54]*2008	…	1.50*** (1.11-2.03)
[45–54]*2010	…	1.54*** (1.13-2.09)
[55–65]*2010	…	1.40** (1.03-1.91)
**Social class**	*(ref = associate profession)*	
Farmer	1.78*** (1.29-2.45)	1.78*** (1.29-2.45)
Craftsman	1.19 (0.91-1.54)	1.18 (0.91-1.54)
Manager	0.89 (0.74-1.08)	0.89 (0.74-1.08)
Office worker	1.19*** (1.05-1.34)	1.18*** (1.05-1.33)
Skilled worker	1.24** (1.01-1.53)	1.24** (1.00-1.52)
Non skilled worker	1.64*** (1.38-1.95)	1.64*** (1.38-1.95)
Inactive	2.31*** (1.85-2.89)	2.29*** (1.84-2.86)
**Marital status**	*(ref = living in a couple)*	
Single	1.82*** (1.61-2.06)	1.82*** (1.60-2.05)
**Complementary health insurance**	*(ref = private)*	
Free	1.54*** (1.33-1.78)	1.54*** (1.33-1.78)
No	2.05*** (1.68-2.51)	2.06*** (1.68-2.52)
	**Health and health care consumption**	
**Chronic disease**	*(ref = no)*	
Yes	1.28*** (1.15-1.43)	1.28*** (1.14-1.42)
**Self-rated health**	*(ref = very good)*	
[0–4] : poor	2.04*** (1.62-2.58)	2.04*** (1.61-2.58)
[5,6] : fair	1.52*** (1.31-1.76)	1.53*** (1.32-1.77)
[7,8]: good	1.21*** (1.08-1.34)	1.21*** (1.09-1.35)
**Nb of consultations with the GP**	*(ref = more then three)*	
Less than two	1.20*** (1.09-1.33)	1.21*** (1.10-1.33)
**Nb of consultations with a specialist**	*(ref = one or more)*	
None	3.43*** (3.11-3.79)	3.43*** (3.11-3.79)
	**Risky behaviours**	
**Tobacco consumption**	*(ref = non smoker)*	
Ex-smoker	0.84*** (0.74-0.94)	0.83*** (0.74-0.94)
Light smoker	0.81*** (0.70-0.92)	0.80*** (0.70-0.92)
Heavy smoker	1.36*** (1.18-1.56)	1.36*** (1.18-1.56)
**Alcohol consumption**	*(ref = safe consumer)*	
Non drinker	1.44*** (1.30-1.59)	1.45*** (1.30-1.60)
Occasionnaly risky consumer	1.09 (0.95-1.25)	1.09 (0.95-1.25)
High risk consumer	1.42*** (1.09-1.85)	1.43*** (1.10-1.86)
**Survey year**	*(ref = 2006)*	
2008	1.02 (0.92-1.13)	0.86 (0.68-1.09)
2010	1.15** (1.03-1.27)	0.90 (0.71-1.15)

Note: The results were adjusted for the region of residence. Nonresponse categories were included in the models, but their coefficients were not reported in the final table. Significance level: *** = 1%; ** = 5%.

All of the variables explained the failure to obtain a test for cervical cancer in the multivariate analyses (N = 13,177 observations used). Compared to younger women (25–34 years), women aged 45–54 years or 55–65 years were more likely not being screened (OR_45–54_ = 1.56 [1.37-1.78], OR_55–65_ = 2.39 [2.08-2.74]. The negative impact of age on cervical screening has increased over time: compared to women aged 25–34 years in 2006, women aged 45–65 years had a higher likelihood of not being screened in 2010. Compared to women having an intermediate profession, living in a couple, and having private complementary health insurance, women who were farmers, office workers, skilled/unskilled workers, or unemployed; who were single; who did not have private health insurance; who had free complementary health insurance (the CMU-C) were significantly more likely to report not being screened. In addition, women who reported having a chronic disease, having good, fair, or poor self-rated health, and using medical care less often (no consultation with a specialist and fewer than two visits to a GP over the past 12 months), were more likely to not have received a screening compared to women who reported greater access to medical care, no chronic disease, and very good self-rated health. Finally, regarding so-called risky lifestyles, the results regarding the probability of not being screened for cervical cancer followed a U-shaped curve: compared to not smoking, being an ex-smoker or a light smoker decreased whereas being a heavy smoker increased the likelihood of not being screened (OR_ex-smoker_ = 0.84 [0.74-0.94], OR_light-smoker_ = 0.81 [0.70-0.92], OR_heavy smoker_ = 1.36 (1.18-1.56). Similarly, compared to safe drinkers, not drinking alcohol or being a high-risk drinker increased the likelihood of not being screened (OR_neverdrink_ = 1.44 [1.30-1.59], OR_highrisk_ = 1.42 [1.09-1.85]). All else being equal, the survey year had a significant impact on whether women reported being screened: women had a higher probability of not being screened for cervical cancer in 2010 than in 2006 (OR_2010_ = 1.15 [1.03-1.27].

### Breast cancer screening

The results of the main-effects model for breast cancer screening are displayed in Table 3 (no interactions were significant at the 5% level).

**Table 3:** **Multivariate logistic regression model of the absence of breast cancer screening (N = 6,229 women)**

Independant variables	No breast cancer screening ***Main effects model***
	OR (95 CI)
	Socioeconomic characteristics
**Age**	*(ref = 50–54)*
[55–59]	0.75*** (0.62-0.89)
[60–64]	0.78*** (0.64-0.94)
[65–74]	1.06 (0.89-1.27)
**Social class**	*(ref = associate profession)*
Farmer	1.08 (0.76-1.55)
Craftsman	1.09 (0.78-1.51)
Manager	1.00 (0.76-1.33)
Office worker	1.18 (0.98-1.42)
Skilled worker	1.28 (0.96-1.70)
Non skilled worker	1.62*** (1.26-2.09)
Inactive	1.61*** (1.14-2.28)
**Marital status**	*(ref = living in a couple)*
Single	1.45*** (1.17-1.80)
**Complementary health insurance**	*(ref = private)*
Free	1.79*** (1.36-2.37)
No	2.08*** (1.57-2.75)
	**Health and health care consumption**
**Chronic disease**	*(ref = no)*
Yes	1.29*** (1.11-1.50)
**Self-rated health**	*(ref = very good)*
[0–4] : poor	1.87*** (1.38-2.54)
[5,6] : fair	1.20 (0.96-1.49)
[7,8]: good	1.06 (0.88-1.28)
**Nb of consultations with the GP**	*(ref = more then three)*
Less than two	1.40*** (1.21-1.62)
**Nb of consultations with a specialist**	*(ref = one or more)*
None	3.00*** (2.59-3.47)
	**Risky behaviours**
**Tobacco consumption**	*(ref = non smoker)*
Ex-smoker	0.92 (0.77-1.10)
Light smoker	1.08 (0.85-1.36)
Heavy smoker	1.84*** (1.44-2.37)
**Alcohol consumption**	*(ref = safe consumer)*
Non drinker	1.27*** (1.09-1.48)
Occasionnaly risky consumer	1.14 (0.90-1.44)
High risk consumer	1.26 (0.88-1.80)
**Survey year**	*(ref = 2006)*
2008	1.16 (1.00-1.35)
2010	1.25*** (1.07-1.46)

Note: The results were adjusted for the region of residence. Nonresponse categories were included in the models, but their coefficients were not reported in the final table. Significance level: *** = 1%; ** = 5%.

For this cancer screening (N = 6,229 observations used), women who reported not being screened more commonly were unskilled worker (OR = 1.62 [1.26-2.09]) or unemployed (OR = 1.61 [1.14-2.28]), single (OR = 1.45 [1.17-1.80]), and did not have a private complementary health insurance (OR = 2.08 [1.57-2.75]) or received free complementary health insurance (OR = 1.79 [1.36-2.37]). In addition, reporting a chronic disease (OR = 1.29 [1.11-1.59]), self-reporting poor health (OR = 1.87 [1.38-2.54]), having a low number of consultations with a GP (OR = 1.40 [1.21-1.62]) or a specialist (OR = 3.00 [2.59-3.47], being a heavy smoker (OR = 1.84 [1.44-2.37]) and never drinking alcohol (OR = 1.84 [1.44-2.37]) significantly increased the probability of not being screened. All else being equal, women were more likely to have not been screened for breast cancer in 2010 than in 2006. We performed a complementary analysis including FOBT uptake in the model and found a significant impact of the variable: all else being equal, not having received FOBT increased the odds ratio associated to not having performed a mammogram by 3.82 [3.18-4.60].

### Colorectal cancer screening

The results of the main-effects and interactions models for colorectal cancer screening are displayed in Table 4 (N = 5,927 observations used for males and N = 6,229 observations used for women).

**Table 4:** **Multivariate logistic regression models of the absence of colorectal cancer screening (N = 5,927 males and N = 6,229 women)**

Independant variables	No colorectal cancer screening ***main effects model***	No colorectal cancer screening ***interactions model***	No colorectal cancer screening ***main effects model***
	Males	Males	Females
	Socioeconomic characteristics		
**Age**	*(ref = 50–54)*		
[55–59]	0.64*** (0.54-0.77)	0.64*** (0.53-0.77)	0.55*** (0.46-0.66)
[60–64]	0.57*** (0.48-0.69)	0.57*** (0.48-0.69)	0.48*** (0.40-0.58)
[65–74]	0.54*** (0.45-0.65)	0.54*** (0.45-0.64)	0.47*** (0.39-0.56)
**Social class**	*(ref = associate profession)*		
Farmer	1.46** (1.08-1.99)	1.46*** (1.07-1.99)	1.06 (0.76-1.50)
Craftsman	1.19 (0.95-1.50)	1.20 (0.95-1.51)	1.22 (0.88-1.68)
Manager	0.99 (0.82-1.19)	0.99 (0.82-1.19)	0.96 (0.75-1.22)
Office worker	1.15 (0.88-1.51)	1.15 (0.88-1.51)	0.87 (0.74-1.03)
Skilled worker	1.08 (0.90-1.29)	1.08 (0.90-1.28)	1.10 (0.83-1.47)
Non skilled worker	1.60*** (1.23-2.08)	1.62*** (1.25-2.11)	1.03 (0.80-1.33)
Inactive	7.18 (0.92-55.82)	6.07 (0.77-47.70)	1.08 (0.75-1.57)
**Marital status**	*(ref = living in a couple)*		
Single	1.12 (0.77-1.62)	1.13 (0.77-1.64)	1.14 (0.90-1.44)
**Complementary health insurance**	*(ref = private)*		
Free	1.18 (0.81-1.72)	0.53** (0.30-0.94)	1.05 (0.76-1.45)
No	1.40 (1.00-1.97)	1.10 (0.64-1.90)	1.20 (0.85-1.70)
Compl. health insurance*survey year	*(ref: compl health insurance = 'private'; survey year = '2008')*		
Free* 2010	…	4.31*** (1.90-9.76)	…
	**Health and health care consumption**		
**Chronic disease**	*(ref = no)*		
Yes	0.95 (0.82-1.10)	0.96 (0.83-1.11)	0.97 (0.84-1.12)
**Self-rated health**	*(ref = very good)*		
[0–4]: poor	1.31 (0.94-1.82)	1.32 (0.95-1.83)	1.48** (1.07-2.03)
[5,6]: fair	1.21 (0.97-1.50)	1.20 (0.97-1.49)	1.17 (0.95-1.45)
[7,8]: good	1.02 (0.86-1.21)	1.03 (0.86-1.22)	1.14 (0.96-1.36)
**Nb of consultations with a GP**	*(ref = more then three)*		
Less then two	1.22*** (1.06-1.41)	1.22*** (1.06-1.41)	1.18** (1.02-1.36)
**Nb of consultations with a specialist**	*(ref = one or more)*		
None	1.29*** (1.12-1.49)	1.30*** (1.12-1.50)	1.68*** (1.45-1.96)
	**Risky behaviours**		
**Tobacco consumption**	*(ref = non smoker)*		
Ex-smoker	1.08 (0.93-1.27)	1.08 (0.93-1.27)	1.00 (0.85-1.17)
Light smoker	1.28** (1.03-1.59)	1.29*** (1.04-1.62)	1.20 (0.94-1.52)
Heavy smoker	1.70*** (1.31-2.22)	1.71*** (1.31-2.23)	1.68*** (1.23-2.28)
**Alcohol consumption**	*(ref = safe consumer)*		
Non drinker	1.19 (0.96-1.48)	1.18 (0.95-1.47)	1.22** (1.05-1.42)
Occasionnaly risky consumer	0.95 (0.82-1.11)	0.95 (0.82-1.11)	1.19 (0.95-1.49)
High risk consumer	1.13 (0.93-1.37)	1.13 (0.93-1.38)	1.44 (0.98-2.09)
**Survey year**	*(ref = 2008)*		
2006	1.15 (0.99-1.35)	1.12 (0.95-1.23)	1.52*** (1.30-1.77)
2010	0.45*** (0.39-0.52)	0.42*** (0.36-0.49)	0.45*** (0.39-0.52)

Note: The results were adjusted for the region of residence. Nonresponse categories were included in the models, but their coefficients were not reported in the final table. Significance level: *** = 1%; ** = 5%.

For the two genders, the determinants of not being screened were being younger (50–54 years of age), being an unskilled worker, having less health care consumption (fewer than 3 visits to a GP or no visits to a specialist over the past twelve months), and being a heavy smoker. All else being equal, the likelihood of not being screened for colorectal cancer was significantly lower in 2010 than in 2008. There were differences regarding the impact of other determinants on FOBT uptake between males and females: females reporting poor health (OR = 1.48 [1.07-2.03]) and never drinking alcohol (OR = 1.22 [1.05-1.42]) had a higher likelihood of not being screened, but it was not the case for males. Conversely, being a light smoker increased the likelihood of not being screened for males (OR = 1.28 [1.03-2.22]) but not for females. One interaction was significant in the model for males: compared to having private complementary insurance, having free complementary health insurance (the CMU-C) was associated with a reduced probability of not being screened for colorectal cancer in 2008 (OR = 0.53 [0.30-0.94]) but a higher probability of not being screened in 2010 (OR = 4.31 [1.90-9.76]). We performed a second analysis for females in which we included whether they had received a mammogram in the past two years into the model and found that this variable had a significant impact: not having received a mammogram increased the odds ratio associated to not having received a FOBT by 3.85 [3.20-4.63].

## Discussion

This study aimed to examine the determinants of and trends in the failure to receive breast, cervical, and colorectal cancer screenings in France between 2006 and 2010, a period in which various incentive policies were implemented. We focused on three types of cancer that were either the focus of recent debate or the subject of mass screening policies that were implemented following national and international guidelines. However, the supply context and incentive policies related to these types of screening differ. A national programme for breast cancer screening has been in place since 2004 and highly publicised for many years. Mammography is generally prescribed by a GP or gynaecologist and performed in a radiology centre. A Pap smear is most often performed by a gynaecologist or GP before it is sent to a laboratory for analysis, but a national programme has not been established (only experiments are ongoing). This test has been the focus of a number of economic evaluations aiming to provide recommendations for the vaccine against human papillomavirus. A national programme for colorectal cancer screening that targets the entire population over the age of 50 years was recently implemented. Colorectal cancer screening is most often prescribed by a GP but may also be recommended by a gastroenterologist or anatomopathologist.

Our results show that the rates of cervical and breast cancer screening use in France decreased between 2006 and 2010. These results, which are consistent with those of other studies obtained in different contexts and countries [21], are particularly disappointing given the maintenance or reinforcement of certain incentive policies (*Cancer Plan 2009–2013*). In contrast, the rate of colorectal cancer screening uptake increased substantially between 2006 and 2010. This result can be explained by the introduction of a national cancer screening programme in 2009 that combined two actions: a voucher mailed to eligible individuals on the demand side and a lump sum payment to GPs on the supply side.

According to our results, young age, low socioeconomic status, and poor or fair self-rated health had the strongest negative impact on cervical screening uptake relative to breast and colorectal cancer screening uptake. This result could be explained by the absence of a national programme for cervical screening and, as a consequence, reduced media attention on cervical screening and/or by the costs associated with cervical screening (i.e., the screening is not free) [22]. Furthermore, the context of cervical screening differs from that of other types of cancer screening, as cervical screening is often provided directly by a gynaecologist during a consultation, which increases inequalities related to access to care. Women who lived in a couple were more likely to consult a gynaecologist for contraceptive treatment and thus were more likely to be screened. In addition, the higher screening uptake for women aged between 25 and 34 years was likely related to consultation for one or several pregnancies, which are associated with more frequent visits to specialists. By extension, the provision of cervical screening may be related to the proximity of a gynaecologist (i.e., living in a more urban area where the density of specialists is much higher). Indeed, having consulted a gynaecologist in the past 12 months constituted a very strong determinant of Pap smear and mammography uptake. We performed separate analyses including gynaecologist visits adjusted for all other covariates and found that this variable had a substantial impact on cervical screening uptake (OR = 7.8 [6.2-9.4]) and a less pronounced impact on mammography screening (OR = 3.5 [2.5-4.7]). These results confirm the need to strengthen efforts to reduce inequalities in access to specialists, especially for cancers that are not the subject of a national programme (e.g., cervical cancer).

Concerning breast cancer screening, socioeconomic characteristics had affected the likelihood of undergoing a mammogram, but the effect was less pronounced than that for cervical screening. A possible explanation for this result is that the combination of highly publicised information campaigns for breast cancer in France and the national screening programme indeed targeted a larger population, but economic inequalities have persisted: not having private complementary health insurance (whether free or not) decreased the probability of screening. In France, not having complementary health insurance and, to a greater extent, receiving free complementary health insurance are often associated with low to very low household income. Moreover, even though mammograms are free of charge in the context of organised cancer screening, lower overall access to the health system (e.g., physician care) may prevent individuals from receiving the test. Thus, even though a national programme was implemented in 2004, i.e., before the study period, inequalities in access to breast cancer screening persist and do not seem to have declined over time.

Regarding colorectal cancer screening, despite the introduction of a national programme in 2009, age-related screening disparities have persisted: individuals under 54 years of age were less likely to have been tested. The complementary health insurance variable had no effect on colorectal cancer screening uptake, in contrast to cervical and breast cancer screening uptake, over the study period. However, among males, inequalities related to having free complementary health insurance (the CMU-C) were significant in 2010. Moreover, individuals with less access to health care (GPs or specialists) were less likely to be tested. Thus, health authorities could intensify efforts to promote increased access to screening for the most disadvantaged populations. For this purpose, a national programme is one mechanism among many and should likely be complemented by targeted primary prevention policies aimed at reducing these inequalities.

Another interesting result of our paper concerns the impact of individual risk behaviours and the use or nonuse of other screening tests. The results for individuals who engaged in high-risk behaviours in terms of alcohol and tobacco consumption were consistent with our expectations and the results of previous studies [23, 24]. One possible rationale for their lower screening uptake could relate to economic trade-offs based on time preferences [25] and/or fear of disease. However, surprisingly, individuals reporting very healthy behaviours (never consuming alcohol, never smoking) also had lower levels of screening uptake. This result is interesting, as it suggests that more targeted primary prevention policies are needed for these individuals, for instance, by combating the notion that cancer only affects individuals with more risky lifestyles.

### Limitations of the study

The main shortcomings of the study are related to the declarative nature of the data. Specifically, so-called social desirability bias may have resulted in an overestimation of screening uptake. In France, the Institute for Public Health Surveillance (INVS) provides an annual estimation of breast cancer screening (since 2004) and colorectal cancer screening (since 2009). Concerning breast cancer, 49.3% of individuals were screened in 2006, 52.5% in 2008 and 52.0% in 2010^e^, and concerning colorectal cancer, 34% of individuals were screened between 2009 and 2010^f^. Thus, the screening rates in the ESPS samples seem to overestimate INVS’s rates (even though they are not comparable for breast cancer screening because the "inclusion" period is not the same), but they are consistent with other self-reported measures provided for instance by the French National Institute for Prevention and Health Education (INPES) [13].

Other determinants of screening, such as cancer-related family history [26], beliefs and knowledge [27–30], or the availability of doctors [31, 32], remained unidentifiable in our study. Other studies have also shown the impact of income on the probability of being screened [18, 33]. Owing to numerous missing values and to avoid increasing the risk of multicollinearity with the health insurance and social class variables, we did not adjust for income.

## Conclusion

Our results demonstrate that persistent obstacles to screening, which are primarily related to socioeconomic characteristics, health care access, and risky behaviours, remain. However, we could not precisely determine if low screening uptake result from lower patient uptake (i.e., patient choice) or from less access to screening services (i.e., system-level barriers). Further studies should more specifically investigate the associations among attitudes/beliefs, individual barriers in access to care, and screening uptake.

Greater coordination between the different actors involved in cancer screening also seems necessary because, in the context of French national programmes—particularly the breast cancer screening programme—the GP or gynaecologist is not always aware of whether their patients have received a mammogram. Some studies argue that the GP’s role should be to coordinate the patient’s various screenings [34, 35]. Indeed, GPs are the most relevant actors in the prescription of colorectal cancer screening [36], and they play an important (advisory) role in breast cancer and cervical screenings [37].

## Endnotes

^a^For a complete description of the authorisation: http://legimobile.fr/fr/cnil/del/aut/2010/2010-003/.

^b^In France, private complementary health insurance is generally purchased to cover reinsurable copayments that are not covered by public health insurance: for any given service, the reimbursement is computed as a percentage of regulated prices, and some providers are also allowed to charge additional fees. Below a certain income threshold, individuals can benefit from a form of free complementary health insurance called the CMU-C. Individuals benefiting from the CMU-C are generally more socially and economically disadvantaged.

^c^We did not include breast cancer and colorectal cancer screening as predictors of cervical cancer screening uptake because the age groups differed.

^d^We thank an anonymous referee for offering two other rationales for this stratification: first other factors affecting colorectal screening may differ by sex (e.g. health consumption, risky behaviours); second, this stratification is useful for examining whether breast cancer screening compliance predicts the likelihood of colorectal screening among women.

^e^http://www.invs.sante.fr/Dossiers-thematiques/Maladies-chroniques-et-traumatismes/Cancers/Evaluation-des-programmes-de-depistage-des-cancers/Evaluation-du-programme-de-depistage-du-cancer-du-sein.

^f^http://www.invs.sante.fr/Dossiers-thematiques/Maladies-chroniques-et-traumatismes/Cancers/Evaluation-des-programmes-de-depistage-des-cancers/Evaluation-du-programme-de-depistage-du-cancer-colorectal.
